# Effects of Covalent Functionalization of MWCNTs on the Thermal Properties and Non-Isothermal Crystallization Behaviors of PPS Composites

**DOI:** 10.3390/polym9100460

**Published:** 2017-09-21

**Authors:** Myounguk Kim, Jungmin Lee, Hyun-gyoo Roh, Dahyun Kim, Juhee Byeon, Jongshin Park

**Affiliations:** 1 Department of Biosystems & Biomaterials Science and Engineering, Seoul National University, Seoul 08826, Korea; myounguk@snu.ac.kr (M.K.); archon04@snu.ac.kr (J.L.); ssangno@snu.ac.kr (H.-g.R.); dahyun39@snu.ac.kr (D.K.); hawaian7@snu.ac.kr (J.B.); 2 Research Institute for Agriculture and Life Sciences, Seoul 08826, Korea

**Keywords:** poly(phenylene sulfide), multi-walled carbon nanotubes, covalent functionalization, thermal property, non-isothermal crystallization behavior

## Abstract

In this study, a PPS/MWCNTs composite was prepared with poly(phenylene sulfide) (PPS), as well as pristine and covalent functionalized multi-walled carbon nanotubes (MWCNTs) via melt-blending techniques. Moreover, the dispersion of the MWCNTs on the PPS matrix was improved by covalent functionalization as can be seen from a Field-Emission Scanning Electron Microscope (FE-SEM) images. The thermal properties of the PPS/MWCNTs composites were characterized using a thermal conductivity analyzer, and a differential scanning calorimeter (DSC). To analyze the crystallization behavior of polymers under conditions similar with those in industry, the non-isothermal crystallization behaviors of the PPS/MWCNTs composites were confirmed using various kinetic equations, such as the modified Avrami equation and Avrami-Ozawa combined equation. The crystallization rate of PPS/1 wt % pristine MWCNTs composite (PPSP1) was faster because of the intrinsic nucleation effect of the MWCNTs. However, the crystallization rates of the composites containing covalently-functionalized MWCNTs were slower than PPSP1 because of the destruction of the MWCNTs graphitic structure via covalent functionalization. Furthermore, the activation energies calculated by Kissinger’s method were consistently decreased by covalent functionalization.

## 1. Introduction

Recently, the use of super-engineering plastics with superior heat resistance and physical properties compared to other polymers has increased. In this study, poly(phenylene sulfide) (PPS), a kind of super-engineering plastics, was used as the matrix of thermal conductive polymer composites. PPS is semi-crystalline, aromatic, and thermoplastic polymer with a high melting point (*T*_m_) of approximately 285 °C, outstanding mechanical properties, thermal stability, excellent flame, and chemical resistance [1,2,3]. Due to its superior properties and relatively low density, PPS could be an alternative to metals for special engineering applications like automotive and structural applications [1]. Although PPS has a high melting point, PPS has some drawbacks, including a low glass temperature (*T*_g_) due to the flexible sulfide linkage between the aromatic rings, low thermal conductivity (approximately 0.30 W/mK), and low impact strength. Further engineering applications of the PPS including automotive parts were limited due to these disadvantages. To overcome these drawbacks, PPS composites can be prepared by blending PPS with other organic/inorganic compounds as fillers [1,2,3,4,5]. 

In this study, multi-walled carbon nanotubes (MWCNTs) were chosen as the inorganic filler to blend with PPS. MWCNTs have excellent mechanical, thermal, and electrical properties because of their high aspect ratio and unique graphitic tubular structure [6,7,8,9]. Due to their superior properties, many studies were conducted regarding the polymer composites having better mechanical, thermal, and electrical properties [10,11,12]. However, MWCNTs tend to aggregate because of strong van der Waals forces and π–π interactions between the individual tubes [13]. To prevent aggregation, surface functionalization of MWCNTs by mechanical, irradiation, and physicochemical methods was studied before [14,15,16]. The covalent functionalization of the MWCNTs was performed and analyzed in our previous study using several chemical treatments, such as acid, hydrogen peroxide, and silane coupling agents [17].

To prepare the polymer composites, the homogeneous dispersion of the thermal conductive filler in the polymer matrix is the most important issue. Several methods have been reported and used, such as powder mixing, solution mixing, and melt mixing, to improve the dispersion of a filler in a polymer matrix [18,19]. Since melt mixing is a simple and conventional method widely used for processing thermoplastic resins, melt mixing was chosen as the preparation method for the PPS/MWCNTs composites in this study. 

To the best of our knowledge, no studies have reported on the thermal properties and non-isothermal crystallization behavior of the PPS composites containing covalent functionalized MWCNTs, as well as pristine MWCNTs. Thermal properties including thermal conductivity, thermal transition characteristics, and thermal stability of the composites are important properties for use. Superior physical and thermal properties of PPS and PPS composites are dependent by their crystallization conditions, so many studies were performed [1,3,20]. Therefore, this study aims to discuss the effect of covalent functionalization via chemical treatment on the dispersion in the PPS matrix and the thermal properties and non-isothermal crystallization behavior of the PPS/MWCNTs composites using several kinetic equations.

## 2. Experimental

### 2.1. Materials

Poly(phenylene sulfide) (PPS) used as the polymer matrix of the composite was purchased in a powder form from Zhejiang NHU Special Materials Co., Ltd., Shaoxing, China. The density of PPS was 1.36 g/cm^3^ at 25 °C. The MWCNTs (CM-130) used in this study were synthesized via a catalytic CVD process. They were purchased from Hanwha Nanotech Co., Ltd., Daejeon, Korea. The MWCNTs purchased consists of 15 multiple walls. The outer diameter ranged from 10 to 15 nm, and the aspect ratio was known as 2 × 10^3^. The purity and density were >90 wt %, and 0.05 g/cm^3^, respectively. Hydrogen peroxide, nitric acid and sulfuric acid were purchased from Junsei Chemical Co., Ltd., Tokyo, Japan. γ-Glycidoxypropyltrimethoxysilane (GPTMS) and ethanol were purchased from Samchun Pure Chemical Co., Ltd., Pyeongtaek, Korea. All PPS and MWCNTs powders were dried in a vacuum oven at 120 °C for 3 h to eliminate moisture and other volatile components prior to the surface modification and composite preparation processes.

### 2.2. Covalent Functionalization of the MWCNTs

Three chemical treatments for MWCNTs were chosen as the covalent functionalization methods to prevent aggregation of MWCNTs in PPS matrix. For H_2_SO_4_/HNO_3_ treatment (denoted as the acid treatment), dried MWCNTs were added to a 65% by volume solution with a 3:1 volume ratio of H_2_SO_4_/HNO_3_ in distilled water. After continuous stirring for 10 h at 80 °C to introduce the carboxyl groups, MWCNTs were filtered, cleaned several times with distilled water, and dried. For hydrogen peroxide treatments, dried MWCNTs were added into a 1:1 volume ratio solution of H_2_O_2_/H_2_O. After continuous stirring for 10 h at 80 °C to introduce the hydroxyl groups, MWCNTs were filtered, cleaned several times with distilled water, and dried (hydrogen peroxide-treated MWCNTs). For silane treatments, GPTMS and hydrogen peroxide treated MWCNTs were added into ethanol and stirred at 80 °C for 5 h, followed by filtering, washing, and drying. For these treatments, GPTMS were attached to the hydroxyl groups of the hydrogen peroxide treated MWCNTs (silane-treated MWCNTs) [17].

### 2.3. Preparation of the PPS/MWCNTs Composites

The PPS/MWCNTs composites with various weight percentages (maximum weight percentage was 1 wt %) of the pristine, acid-treated, hydrogen peroxide-treated, and silane-treated MWCNTs were prepared via a melt blending technique. The pre-weighed covalently functionalized MWCNTs and PPS powders were poured into absolute ethanol. The mixture was sonicated for 5 min in a sonication bath for pre-mixing of the feed of melt mixing and then dried in a vacuum oven at 120 °C until all moisture and solvents evaporated. Then, the PPS/MWCNTs mixture was melt-blended at 310 °C using a micro-extruder (Wellzoom, Seoul, Korea) and then compressed using a hot press technique with a two-post manual hydraulic press (#2699, Carver Inc., Wabash, IN, USA) at 290 °C under 20 MPa. Finally, the mold was cooled in the air to room temperature until the sample hardened. The sample codes of the PPS/MWCNTs composites were assigned according with the rule shown below.

Assignation Rule:

PPS + MWCNTs type (P, A, HP, and S) + weight percentage of the MWCNTs.

For the MWCNTs type in the assignation rule, the codes P, A, HP, and S represent the pristine, acid-treated, hydrogen peroxide-treated, and silane-treated MWCNTs, respectively.

### 2.4. Characterization

A spectrum One Fourier Transform Infrared spectrophotometer (Perkin Elmer, Shelton, CA, USA) was used in transmission mode to observe the compatibility between PPS and the covalently functionalized MWCNTs. The spectra obtained ranged between 400 and 4000 cm^−1^ at a resolution of 4 cm^−1^. Field-Emission Scanning Electron Microscopy (FE-SEM, SU70, Hitachi, Tokyo, Japan) was used to observe the dispersion of the MWCNTs on the PPS matrix. Before the FE-SEM characterization, the fractured surface of the PPS/MWCNTs composites was sputter coated with a thin layer of platinum. The thermal conductivity of the PPS/MWCNTs composite was measured using a C-therm TCI thermal conductivity analyzer following the modified transient plane source method (ASTM D7984) at room temperature under normal atmosphere. The measured testing value was averaged five times for each sample. The other properties were measured using 1 wt % of the PPS/MWCNTs composite with the highest thermal conductivity. Field-Emission Transmission Electron Microscopy (FE-TEM, G2 F30 S-TWIN, Tecnai, Eindhoven, Holland) was used to observe graphitic structures of the covalently functionalized MWCNTs. A thermogravimetric analyzer (Q-5000 IR, TA Instruments, Inc., New Castle, DE, USA) was used to observe the changes in the thermal stabilities of the PPS/MWCNTs composites with chemical treatments compared to the neat PPS composites. The PPS/MWCNTs composites were heated from room temperature to 800 °C at a heating rate of 10 °C/min under a nitrogen atmosphere. A differential scanning calorimeter (DSC-Q1000, TA Instruments, Inc., New Castle, DE, USA) was used to observe the changes in the glass, crystallization and melting temperatures of the PPS/MWCNTs composites. Approximately 12 mg of the sample was heated to 310 °C at 10 °C/min and held there for 5 min to eliminate the thermal history. Then, the sample was cooled to room temperature at 10 °C/min. The second heating run was performed in the same manner as the first heating run. Moreover, the non-isothermal crystallization kinetics were characterized as having different cooling rates of 10, 20, and 30 °C/min. The non-isothermal crystallization behaviors of the PPS/MWCNTs composites were analyzed using different kinetic models.

## 3. Results and Discussion

### 3.1. Characterization of the PPS/MWCNTs Composites

**Figure 1:**
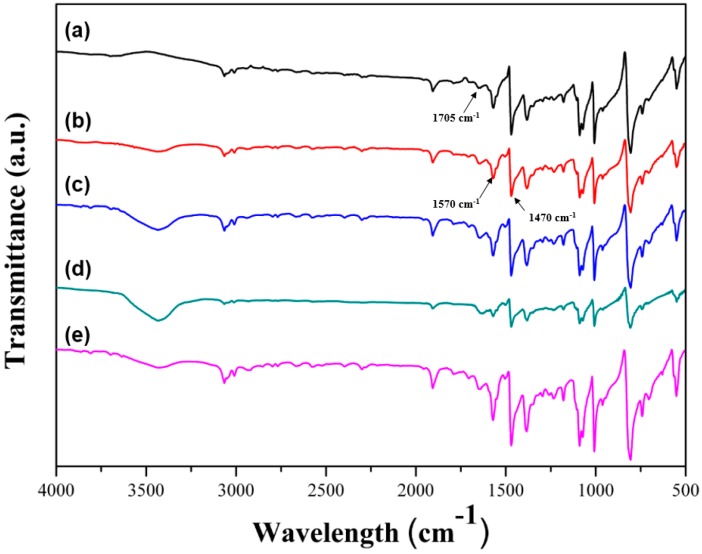
Fourier Transform Infrared spectra of the PPS/MWCNTs composites. (**a**) Neat PPS, (**b**) PPSP1, (**c**) PPSA1, (**d**) PPSHP1, and (**e**) PPSS1.

**Figure 2:**
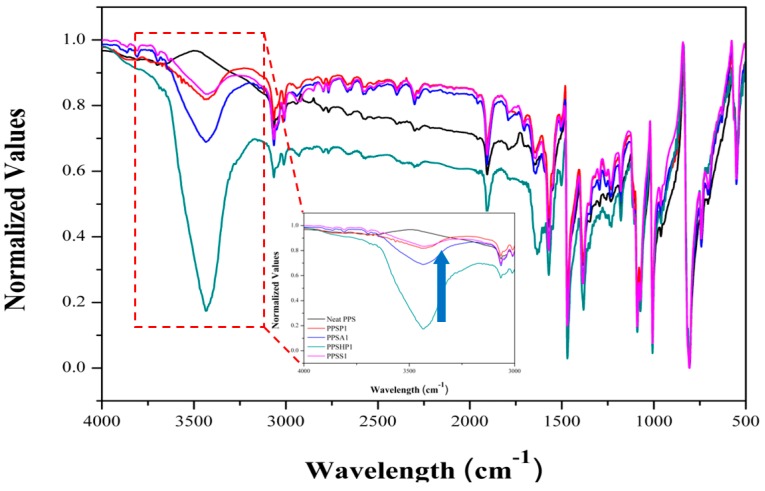
Normalized FT-IR spectra of the PPS/MWCNTs composites (the inset image is an enlarged spectra of the PPS/MWCNTs composites ranged from 3000 to 4000 cm^−1^).

The PPS/MWCNTs composites were characterized to confirm the mixing and dispersion of the MWCNTs on the PPS matrix using FT-IR and SEM. The FT-IR spectra of the PPS/MWCNTs composites are shown in Figure 1. For the FT-IR spectrum of the PPS, there are several characteristic peaks. Two peaks were observed at 1470 and 1570 cm^−1^ and were attributed to symmetric benzene ring stretches. Two other peaks were observed at 1082 and 1092 cm^−1^, which were attributed to asymmetric benzene ring sulfur stretches. For the PPSP1, no significant change in the FT-IR spectrum was observed upon introduction of the MWCNTs. On the other hand, for PPSA1 and PPSHP1, hydroxyl groups or carboxyl groups were observed at 3435 or 1705 cm^−1^. Likewise, for the PPSS1, peak for hydroxyl groups were also observed, and the spectrum was more complicated because of the attached silane coupling agents. The existence of dispersed pristine or covalent functionalized MWCNTs within the PPS matrix was confirmed. Moreover, the normalized FT-IR spectra of all PPS/MWCNTs composites is shown in Figure 2. The inset spectrum is an enlarged spectra ranging from 3000 to 4000 cm^−1^ of the peak attributed to hydroxyl groups by covalent functionalization. The normalized intensity of the peak of the hydroxyl group was significantly decreased by silane treatment because the silane coupling agent reacted with the hydroxyl groups on the surface of the hydrogen peroxide-treated MWCNTs. These data also consistently describe all kinds of the covalently-functionalized MWCNTs that were mixed well with the PPS via the melt-blending process.

**Figure 3:**
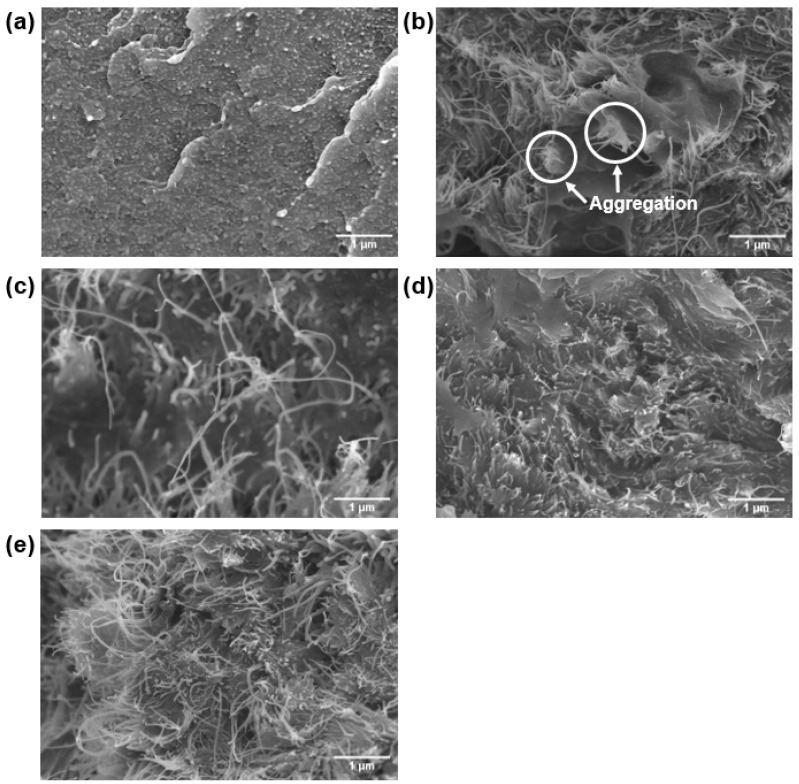
Field-Emission Scanning Electron Microscopy images of the PPS/MWCNTs composites. (**a**) Neat PPS, (**b**) PPSP1, (**c**) PPSA1, (**d**) PPSHP1, and (**e**) PPSS1.

Figure 3 shows the FE-SEM images of the fractured surface of the PPS/MWCNTs composites. For the neat PPS, a smooth and compact surface was observed because of the brittleness of the PPS. For the PPSP1, some aggregates were observed because of the strong van der Waals forces and π–π interactions between the individual tubes. For the PPS/MWCNTs composites containing covalently-functionalized MWCNTs (PPSA1, PPSHP1, and PPSS1), the covalent functionalization led to better dispersion of the MWCNTs on the PPS matrix compared to the PPSP1. The better dispersion of the MWCNTs improved the thermal conductivity and thermal stability of the PPS/MWCNTs composites.

### 3.2. Thermal Properties of the PPS/MWCNTs Composites

**Figure 4:**
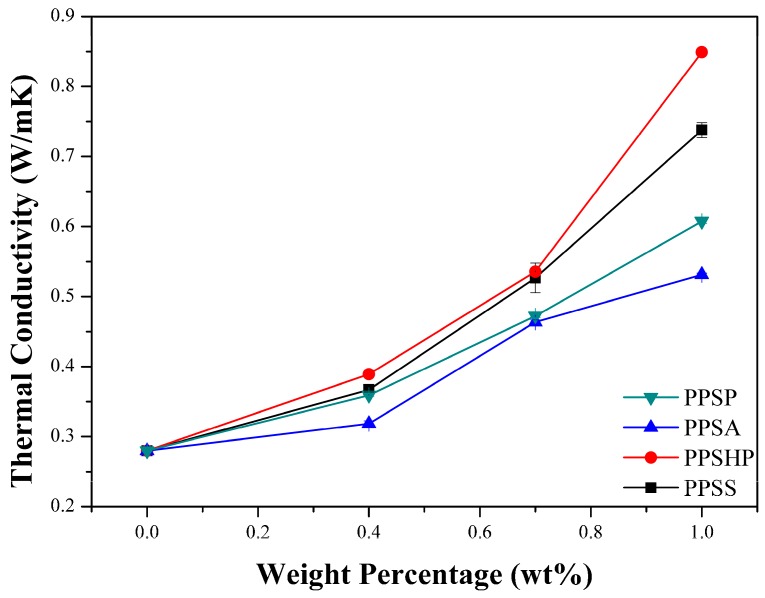
Thermal conductivity of the PPS/MWCNTs composites.

The thermal conductivity in polymeric materials comes from propagating the lattice vibrations generated by a phonon within the material due to interatomic interactions [21,22]. However, the phonon propagating through the material is scattered by thermal resistance, which results in phonon-phonon scattering, boundary scattering, and defect or impurity scattering. Therefore, phonon scattering should be minimized to improve the thermal conductivity [21,23]. To move a phonon easily within a material, the heat transfer path is very important. When fillers are introduced into a polymer matrix, the volume fraction, shape, particle size, and aspect ratio are important factors affecting the thermal conductivity [21,23,24]. The thermal conductivity values of the PPS/MWCNTs composites are shown in Figure 4. The thermal conductivity values of the PPS/MWCNTs composites increased by up to 217%, 190%, 304%, and 264%, respectively, compared to that of neat PPS (0.28 W/mK) and in proportion to the MWCNTs contents, regardless of the chemical treatment. For the PPSA composites, however, the thermal conductivity value was lower than that of the composite containing pristine MWCNTs because of a reduction in the aspect ratio of the MWCNTs from the acid treatment even though the dispersion of the MWCNTs improved shown in Figure 3c. However, the PPSHP showed a higher thermal conductivity than the other PPS/MWCNTs composites because of the enhanced dispersion of the MWCNTs in the PPS matrix shown in Figure 3d and the hydrogen bonding between the PPS matrix and hydrogen peroxide-treated MWCNTs. The interactions between the PPS and functional groups, such as the hydroxyl and carboxyl groups of the MWCNTs, were reported by Zhang et al. [25]. For the PPS/MWCNTs composites containing the silane-treated MWCNTs, the thermal conductivity value was higher than that of the composite containing pristine MWCNTs because of an improvement in the interfacial interaction between the PPS and MWCNTs from attaching the silane coupling agent [26,27]. However, the PPSS composites have a lower thermal conductivity value than that of the PPSHP composites because the silane layers interrupt the hydrogen bonding between PPS and the hydroxyl groups of the MWCNTs. This phenomenon was consistent with the normalized FT-IR spectra of the PPS/MWCNTs composites shown in Figure 2, observing that the intensity of the hydroxyl groups peak was significantly decreased. This data represents the attached silane-coupling agent blocked the hydrogen bonding between the PPS matrix and hydroxyl groups of the MWCNTs.

**Figure 5:**
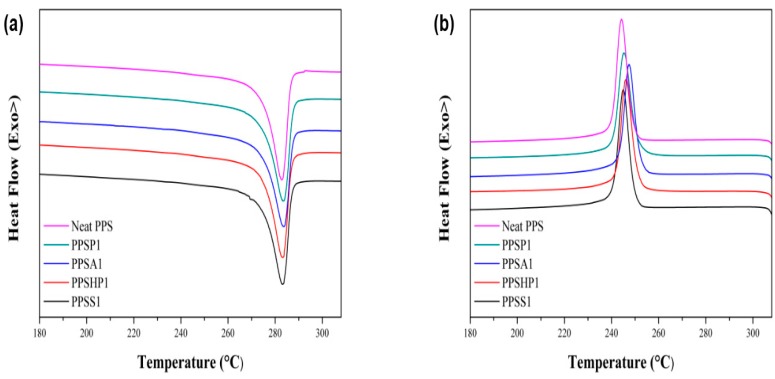
Differential Scanning Calorimetry (**a**) melting curve and (**b**) cooling curve for 10 °C/min heating rate of the PPS/MWCNTs composites.

**Figure 6:**
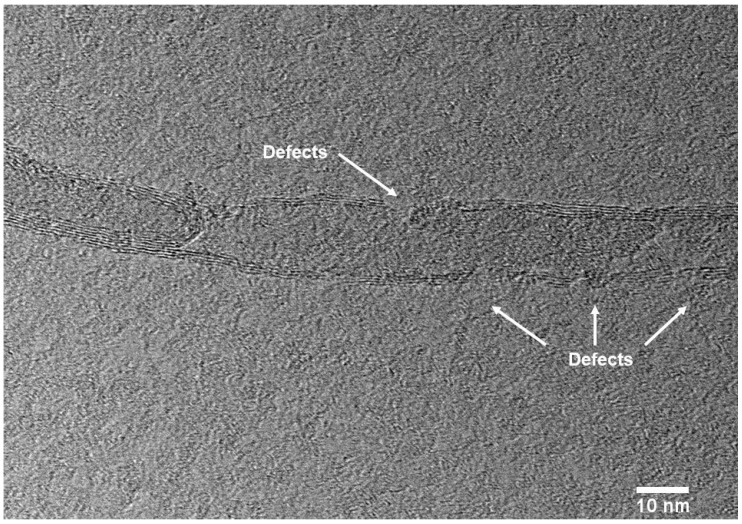
Field-Emission Transmission Electron Microscopy of hydrogen peroxide-treated MWCNTs.

Figure 5 shows the DSC melting and cooling curves of the PPS/MWCNTs composite. Likewise, the DSC characteristic thermal data of the PPS/MWCNTs composites are shown in Table 1. Due to the introduction of pristine and covalently-functionalized MWCNTs into the PPS matrix, the crystallization temperature ($T_{c}$), melting temperature ($T_{m}$) and crystallinity ($\chi_{c}$) also improved upon the introduction of pristine and covalently-functionalized MWCNTs into the PPS matrix. The crystallinity was calculated using Equation (1):

(1) $$\chi_{c}=\frac{\Delta H_{f}}{w\times \Delta H_{f}^{0}}$$

where *w*, $\Delta H_{f}$, and $\Delta H_{f}^{0}$ represent the weight percentage of the PPS, the heat of fusion at the melting point of the PPS/MWCNTs composites, and the heat of fusion of the crystalline PPS, respectively. The heat of fusion of crystalline PPS is 76.5 J/g [28]. Since the MWCNTs act as nucleation agents, many studies have reported that MWCNTs can promote the crystallization via the nucleation effect [29,30,31]. The experimental data were consistent with the reports on the nucleation effect of the MWCNTs. However, for the PPS/MWCNTs composites containing covalently-functionalized MWCNTs (PPSA1, PPSHP1, and PPSS1), the graphitic structures of the MWCNTs were destroyed as the functional groups were attached, which can be estimated from FE-TEM image. Figure 6 shows the defects of graphitic structures of hydrogen peroxide-treated MWCNT. Then, the nucleation effect decreased, which led the decreased crystallinity compared to the PPSP1. 

### 3.3. Non-Isothermal Crystallization Behavior of the PPS/MWCNTs Composites

**Figure 7:**
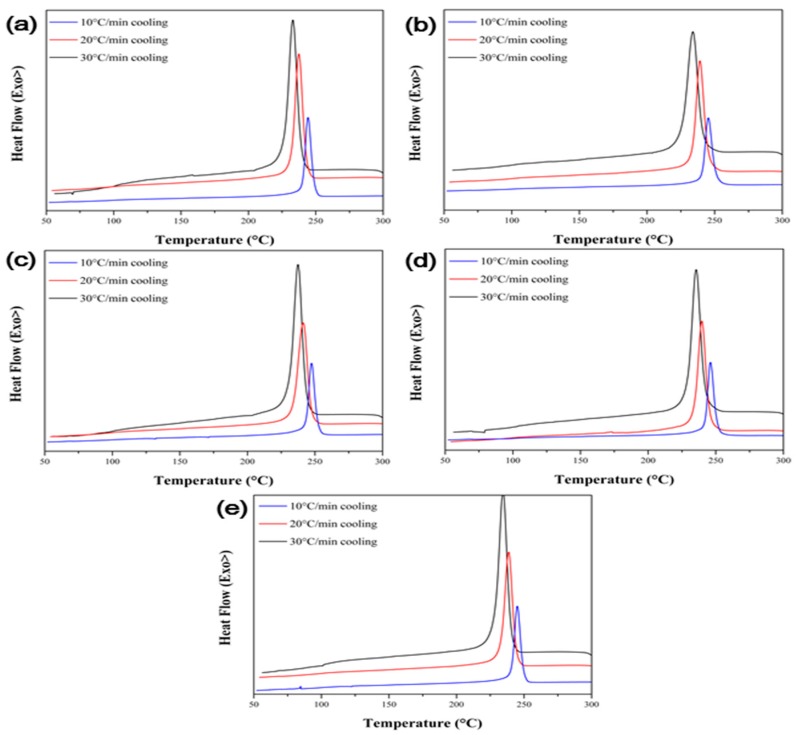
Non-isothermal crystallization cooling curve of the PPS/MWCNTs composites. (**a**) Neat PPS, (**b**) PPSP1, (**c**) PPSA1, (**d**) PPSHP1, and (**e**) PPSS1.

**Figure 8:**
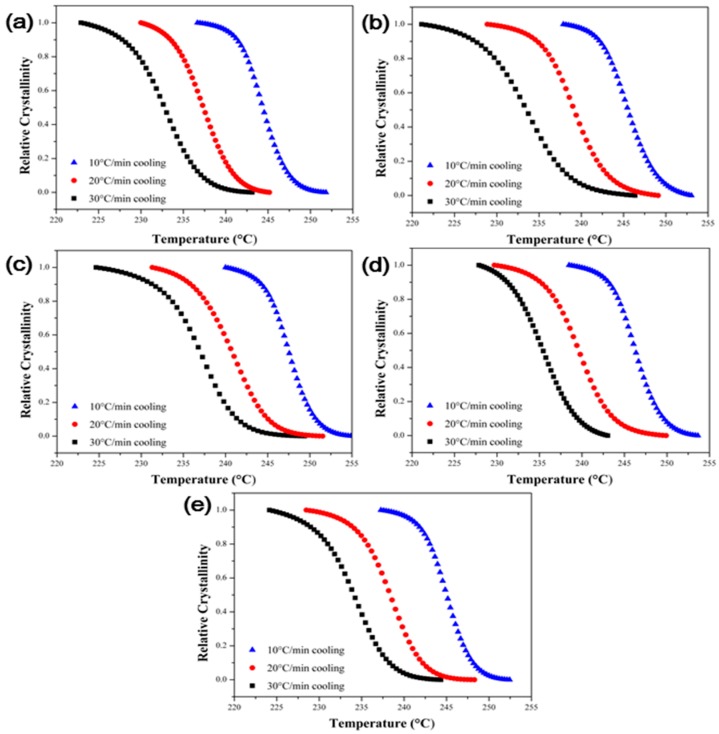
Plots of relative crystallinity versus temperature of the PPS/MWCNTs composites at different cooling rates. (**a**) Neat PPS, (**b**) PPSP1, (**c**) PPSA1, (**d**) PPSHP1, and (**e**) PPSS1.

**Figure 9:**
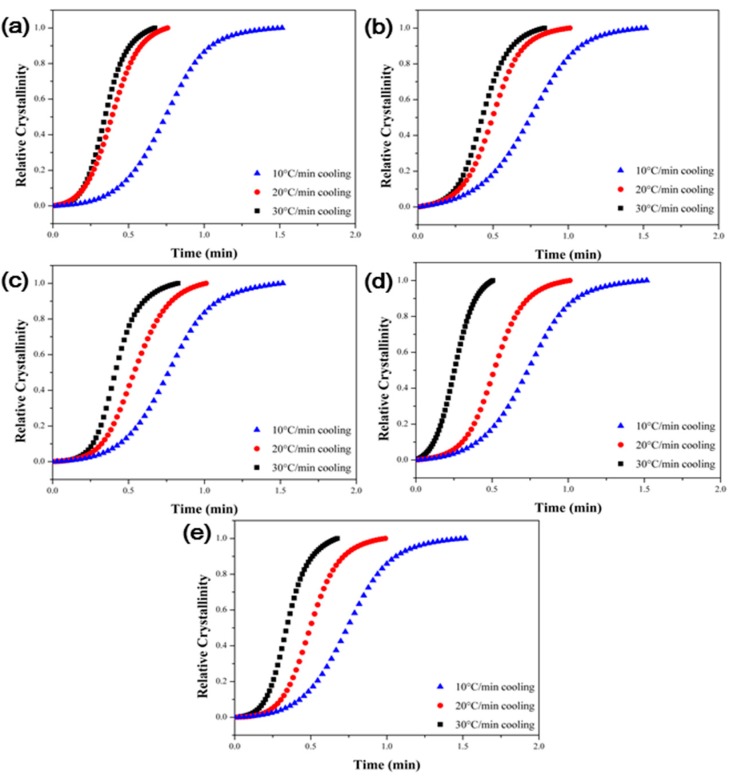
Plots of relative crystallinity versus time of the PPS/MWCNTs composites at different cooling rates. (**a**) Neat PPS, (**b**) PPSP1, (**c**) PPSA1, (**d**) PPSHP1, and (**e**) PPSS1.

The non-isothermal crystallization behavior of the polymer composites is useful to analyze the crystallization behavior under conditions similar with those in actual industrial processes. Figure 7 shows the non-isothermal cooling curve of the PPS/MWCNTs composites under different cooling rates. To analyze the non-isothermal crystallization behavior, the relative crystallinity ($X_{T}$), which is a function of temperature, was calculated using Equation (2) shown below [32,33]:

(2) $$X_{T}=\frac{\int_{T_{i}}^{T}\left(\frac{dH_{c}}{dT}\right)dT}{\int_{T_{i}}^{T_{f}}\left(\frac{dH_{c}}{dT}\right)dT}$$

where $dH_{c}$ represents the crystallization enthalpy measured in non-isothermal conditions, and $T_{i}$, $T_{f}$, and T represent the initial, final, and arbitrary temperatures of the samples during non-isothermal crystallization conditions, respectively. Figure 8 shows the calculated relative crystallinity with the temperature. Moreover, the relative crystallinity has a relationship shown in Equation (3) between the crystallization temperature and time:

(3) $$t=\frac{T-T_{i}}{v}$$

where v is the cooling rate in non-isothermal crystallization condition. Equation (2) can be converted into Equation (4) using Equation (3):

(4) $$X_{t}=\frac{\int_{t_{i}}^{t}\left(\frac{dH_{c}}{dt}\right)dt}{\int_{t_{i}}^{t_{f}}\left(\frac{dH_{c}}{dt}\right)dt}$$

where $t_{i}$, $t_{f}$, and t represent the initial, final, and arbitrary temperatures of the samples during non-isothermal crystallization conditions, respectively. The relative crystallinity with the time is shown in Figure 9.

Several methods are used to analyze the non-isothermal crystallization of polymer composites. Jeziorny analyzed the non-isothermal crystallization behavior by correcting the crystallization rate constant ($Z_{t}$) via modification of the cooling rate ν in the Avrami equation, which is used to analyze isothermal crystallization behavior [34]. The modified Avrami equation is shown in Equation (5):

$$log[-ln\left(1-X_{t}\right)]=logZ_{c}+nlogt$$

(5) $$logZ_{c}=\;\frac{logZ_{t}}{v}$$

**Figure 10:**
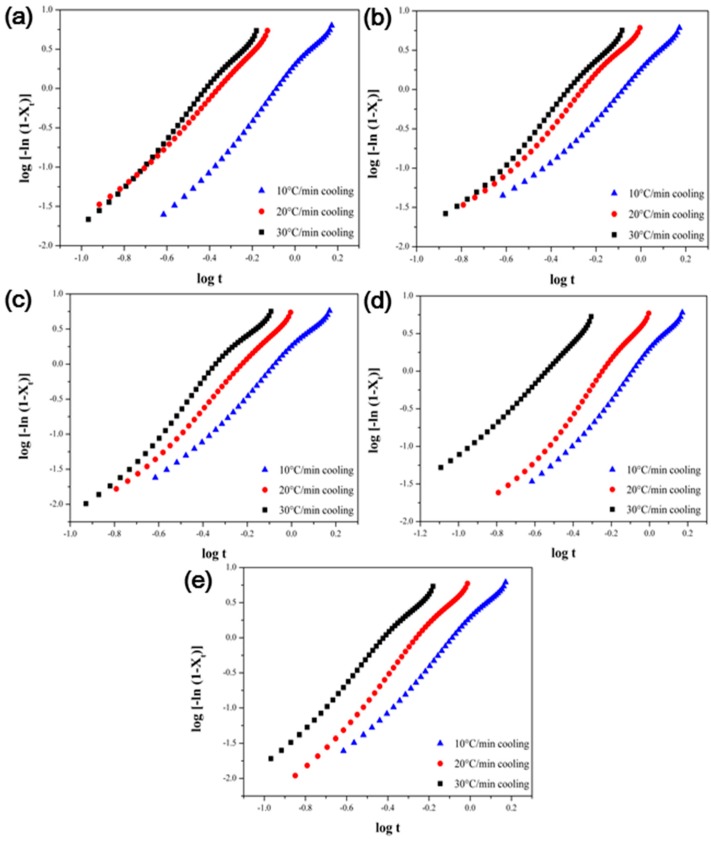
Avrami plots of $\ln[-\ln\left(1-X_{t}\right)]\;$ versus ln *t* for non-isothermal crystallization of the PPS/MWCNTs composites. (**a**) Neat PPS, (**b**) PPSP1, (**c**) PPSA1, (**d**) PPSHP1, and (**e**) PPSS1.

The Avrami constant and crystallization rate constant are determined by plotting the relative crystallinity versus time. The slope and intercept represent the Avrami constant and crystallization rate constant, respectively. The Avrami constant represents the growth of crystals and the mechanism of nucleation. Plots of the relative crystallinity versus time, the Avrami constant and the crystallization rate constant of the PPS/MWCNTs composites are shown in Figure 10 and Table 2. The crystallization mechanism of the PPS did not change significantly with the introduction of pristine or covalently-functionalized MWCNTs. Moreover, the crystallization rate constant increases with the increasing cooling rate v.

Ozawa analyzed the non-isothermal crystallization behavior by modifying the Avrami equation at constant cooling rate conditions. The Ozawa method assumes that the non-isothermal crystallization process is composed of large numbers of small infinitesimal isothermal steps. The Ozawa equation represents the relative crystallinity as a function of the constant cooling rate shown in Equation (6) [32,35,36,37]:

(6) $$\;log[-ln\left(1-X_{T}\right)]=logK\left(T\right)-mlogv$$

where K(T) represents the kinetic parameter associated with temperature, T, and m also represent the Ozawa exponent related to the mechanism of nucleation and crystal growth. However, the Ozawa method is not suitable for analyzing the non-isothermal behavior of the various polymer composites because it does not consider that both the time and cooling rate could be responsible for the experimental data differences, which indicates the varying physical states of the system [32,33,36].

**Figure 11:**
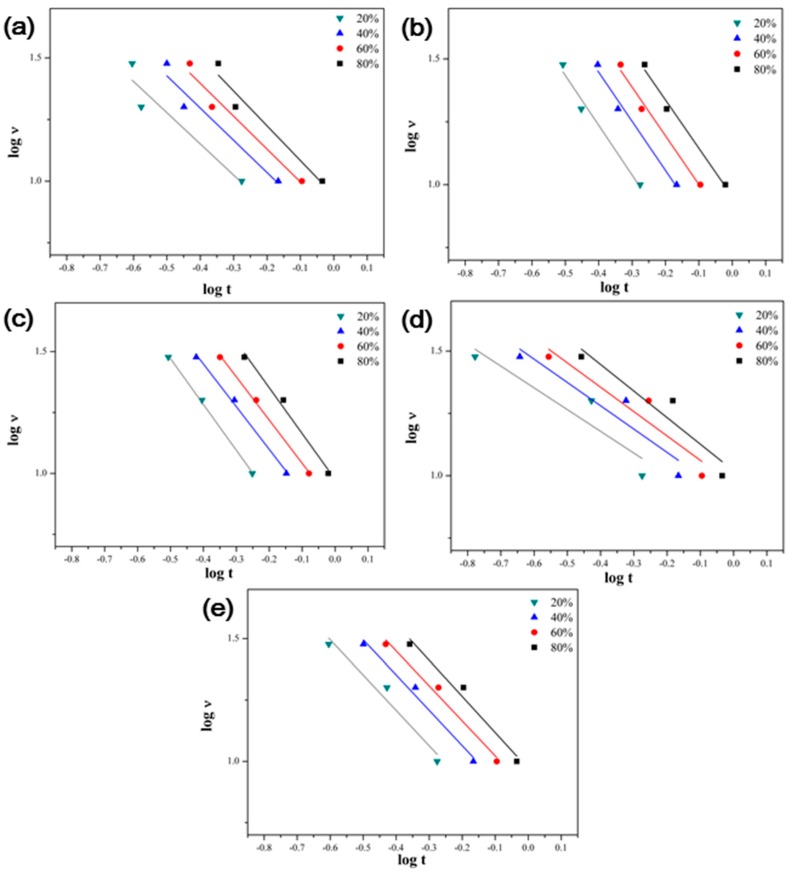
Plots of *log* v versus *log t* of the PPS/MWCNTs composites at different relative crystallinity. (**a**) Neat PPS, (**b**) PPSP1, (**c**) PPSA1, (**d**) PPSHP1, and (**e**) PPSS1.

Liu analyzed the non-isothermal crystallization behavior with time and the cooling rate at a specific relative crystallinity by combining the Avrami equation with the Ozawa equation to overcome the problems of the Ozawa equation [38]. The Avrami-Ozawa combined equation is shown in Equation (7):

(7)  logv=logF(T)−αlogt

where F(T) represents the value of the crystallization rate when the system has a certain relative crystallinity, which is calculated from ${\left[K\left(T\right)/Z_{t}\right]}^{1/m}$. If the F(T) value is smaller, the crystallization rate is higher. 𝛼 is the ratio of the Avrami exponent, n, to the Ozawa exponent, m. Plots of the cooling rate versus time, the F(T) and 𝛼 values of the PPS/MWCNTs composites are shown in Figure 11 and Table 3. The F(T) values for the PPSP1 composites were lower than that of the neat PPS. This is because the heterogeneous crystallization rate increased due to the nucleation effect of the MWCNTs. However, for the composites containing covalently-functionalized MWCNTs (PPSA1, PPSHP1, and PPSS1), the heterogeneous crystallization rate decreased compared to the composite with the pristine MWCNTs because the covalent functionalization destroyed the graphitic structure of the MWCNTs, which resulted in a decrease in the nucleation sites [39].

### 3.4. Activation Energy of the PPS/MWCNTs Composites

**Figure 12:**
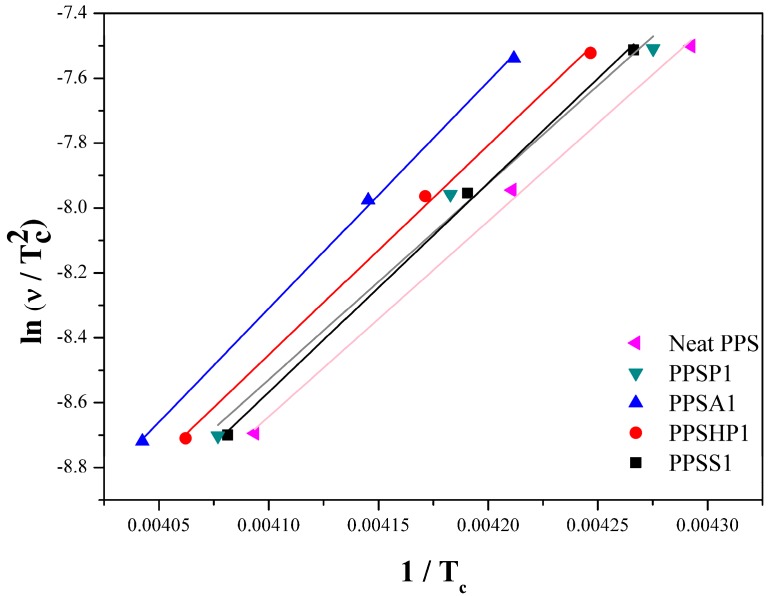
Kissinger plots of $\ln\;\left(v/T_{c}^{2}\right)\;$ versus $1/T_{c}\;$ of the PPS/MWCNTs composites.

The activation energy of the PPS/MWCNTs composites under non-isothermal crystallization can also be characterized based on the crystallization temperature, $T_{c}$, at the cooling rate v using the Kissinger method shown in Equation (8) [32,36,40]:

(8) $$\frac{d\left[ln\frac{v}{T_{c}^{2}}\right]}{d\left(\frac{1}{T_{c}}\right)}=-\frac{E_{c}}{R}$$

where *R* represents a gas constant, which is 8.314 J/mol·K. The activation energy can be obtained by multiplying the slope of Equation (8) by the gas constant. The Kissinger plot and the activation energy of the PPS/MWCNTs composites are shown in Figure 12, and Table 4. For PPSP1, the pristine MWCNTs aggregated on the PPS matrix shown in Figure 2, and the activation energy slightly increased compared to the neat PPS. However, for the composites containing the covalently-functionalized MWCNTs (PPSA1, PPSHP1, and PPSS1), the activation energy considerably increased because of the decrease in the nucleation sites due to covalent functionalization. This means that more energy is needed to achieve crystallization. In short, it is difficult for crystallization to occur in the composites containing the covalently-functionalized MWCNTs.

## 4. Conclusions

Better dispersion was observed on the PPS matrix in the FE-SEM images with the covalently-functionalized MWCNTs compared to the pristine MWCNTs. The better MWCNT dispersion led to higher thermal conductivity, melting temperature, and crystallinity of the PPS/MWCNTs composites compared to the neat PPS. However, for the PPSA composites, the aspect ratio of the MWCNTs decreased because of the acid treatment. This resulted in a lower thermal conductivity than neat PPS. The non-isothermal crystallization behavior of the PPS/MWCNTs composite was analyzed using various kinetic equations. For the non-isothermal crystallization behavior analyzed using the modified Avrami equation, the crystallization mechanism of the PPS/MWCNTs composite did not change significantly with the introduction of pristine or covalently-functionalized MWCNTs. Moreover, for the non-isothermal crystallization behavior analyzed using the Avrami-Ozawa combined equation, the crystallization rate of the PPS/MWCNTs composites was faster because of the nucleation effect of the MWCNTs. However, the crystallization rates of the PPS/MWCNTs composites containing the covalently-functionalized MWCNTs decrease due to the destruction of the graphitic structure of the MWCNTs via covalent functionalization in the case of PPSA1, PPSHP1, and PPSS1. The activation energy calculated using the Kissinger method was also consistent with the non-isothermal crystallization behavior of the PPS/MWCNTs composites.

## Figures and Tables

**Table 1:** DSC thermal data of the PPS/MWCNTs composites.

Sample	$T_{c}\;\left({}^{\circ}C\right)$	ΔHc (Jg)	$T_{m}\;\left({}^{\circ}C\right)$	ΔHf (Jg)	$\chi_{c}\;\left(\%\right)$
Neat PPS	244.28	45.97	282.84	37.06	48.44
PPSP1	245.29	46.15	283.57	41.77	55.15
PPSA1	247.37	43.79	283.62	40.70	53.74
PPSHP1	246.18	45.22	283.31	40.81	53.89
PPSS1	245.02	46.00	283.19	39.10	51.63

**Table 2:** Non-isothermal kinetic parameters of the PPS/MWCNTs composites at different cooling rates using the modified Avrami equation.

Cooling Rate (°C/min)	Neat PPS		PPSP1		PPSA1		PPSHP1		PPSS1	
	n	$Z_{c}$	n	$Z_{c}$	n	$Z_{c}$	n	$Z_{c}$	n	$Z_{c}$
10	3.19	1.06	2.84	1.06	3.16	1.05	3.00	1.06	3.17	1.06
20	2.87	1.13	3.03	1.09	3.31	1.09	3.20	1.10	3.39	1.10
30	3.14	1.10	3.11	1.08	3.46	1.09	2.57	1.11	3.19	1.10

**Table 3:** Non-isothermal kinetic parameters of the PPS/MWCNTs composites at different relative crystallinity using the Avrami-Ozawa combined equation.

Relative Crystallinity (%)	Neat PPS		PPSP1		PPSA1		PPSHP1		PPSS1	
	α	*F(T)*	α	*F(T)*	α	*F(T)*	α	*F(T)*	α	*F(T)*
20	1.27	4.41	1.98	2.78	1.88	3.40	0.87	6.75	1.44	4.28
40	1.31	5.93	1.95	4.64	1.75	5.60	0.93	8.06	1.44	5.98
60	1.33	7.29	1.94	6.41	1.77	7.31	0.98	9.20	1.42	7.59
80	1.41	8.76	1.92	8.97	1.88	9.45	1.07	10.47	1.47	9.34

**Table 4:** The activation energy of non-isothermal crystallization of the PPS/MWCNTs composites.

Sample	Neat PPS	PPSP1	PPSA1	PPSHP1	PPSS1
$E_{c}$ (kJ/mol)	50.13	50.24	58.19	53.73	53.60
